# Remediation of soils on municipal rendering plant territories using *Miscanthus* × *giganteus*

**DOI:** 10.1007/s11356-022-23724-z

**Published:** 2022-10-26

**Authors:** Anna Grzegórska, Natalia Czaplicka, Jacek Antonkiewicz, Piotr Rybarczyk, Agnieszka Baran, Krzysztof Dobrzyński, Dawid Zabrocki, Andrzej Rogala

**Affiliations:** 1 Department of Process Engineering and Chemical Technology, Faculty of Chemistry, Gdansk University of Technology, Narutowicza 11/12, 80-233 Gdansk, Poland; 2 Department of Agricultural and Environmental Chemistry, Faculty of Agriculture and Economics, University of Agriculture in Krakow, Av. Mickiewicza 21, 31-120 Krakow, Poland; 3 Rendering Plant in Gdańsk, Zakład Utylizacyjny Sp. z o.o. w, Jabłoniowa 55, 80-180 Gdansk, Poland; 4 Research and Development Dawid Zabrocki, Jęczniki Wielkie 36A, 77-300 Czluchow, Poland

**Keywords:** Contaminated soil, Energy crops, Heavy metals, *Miscanthus* × *giganteus*, Phytoremediation, Soil improver

## Abstract

Phytoremediation, as a cost-effective, highly efficient, environmentally friendly, and green approach, gained attention to the removal of metals, including heavy metals, from contaminated soils. The toxic nature of heavy metals can have an adverse effect on human health and the ecosystem, and their removal remains a worldwide problem. Therefore, in this study, a field experiment was carried out to evaluate the potential of *Miscanthus* × *giganteus* for the removal of ten microelements and heavy metals (Al, Zn, Fe, Pb, Cd, Co, Cr, Cu, Mn, Ni) from contaminated soil in the territory of a Municipal Waste Rendering Plant. Moreover, the effect of the incorporation of soil improver obtained upon composting biodegradable waste as well as the addition of highly contaminated post-industrial soil on the efficiency of phytoremediation and plant growth was described. The soil improver (SK-8) was applied to the soil at a rate of 200 Mg ha^−1^ and 400 Mg‧ha^−1^. Meanwhile, in the last object, 100 Mg‧ha^−1^ of highly contaminated post-industrial soil was added. Herein, the research was aimed at assessing the possibility of phytoextraction of heavy metals from soils with different physicochemical properties. The results showed that plants cultivated in soil with 400 Mg‧ha^−1^ of soil improver exhibited the highest yield (approximately 85% mass increase compared to the soil without additives). Furthermore, the application of a single dose of SK-8 (200 Mg ha^−1^) increased the uptake of Al, Fe, Co, Pb, Mn, Ni, and Cd by *Miscanthus* × *giganteus* compared to the soil without additives. Additionally, the performed biotests demonstrated no or low toxicity of the investigated soils affecting the test organisms. However, in all experiments, the phytorecovery of the elements did not exceed 1% of the amount introduced to the soil, which may result from a short cultivation period and large doses of SK-8 or highly contaminated post-industrial soil.

## Introduction

Environmental pollution is growing rapidly around the world. Heavy metals, pesticides, plasticizers, surfactants, pharmaceuticals, and dyes have been commonly detected in many compartments, such as soil, groundwater or surface water, and air (Hilili et al. 2021; Singh et al. 2021; Supreeth 2021; Wieczorek et al. 2021). Many of them are not easily biodegradable and therefore possess long-term persistence in ecosystems (Alharbi et al. 2018; Kallawar et al. 2021; Zhang et al. 2020). High levels of pollutants can have adverse effects on the environment, natural habitat, and human health (Konduracka, 2019). Therefore, appropriate treatment methods are still being developed.

One of the inexpensive, simple, and sustainable techniques to remove harmful contaminants from various compartments of the environment is phytoremediation (Yan et al. 2021; Yang et al. 2020). This in situ approach involves green plants to clean the soil, aquatic systems, or air (Farraji et al. 2020). Generally, it is based on the ability of plants to uptake, accumulate, and stabilize pollutants (Pathak et al. 2020). Some of them may be transformed into simpler and less toxic compounds (Shrirangasami et al. 2020). In addition, biomass after phytoremediation processes can be converted into valuable bioenergy sources (Grzegórska et al. 2020). However, one of the main limitations of the phytoremediation is the slow growth of plants, making it a time-consuming process (Tiwari et al. 2022).

Depending on the type of environment and pollutants, various processes can be identified, thanks to which these pollutants are stabilized or degraded and removed from the site. The main groups of such processes include phytostabilization (consists in retaining the contaminants in the soil to prevent their further dispersion in the environment), phytodegradation (degradation of organic compounds by root enzymes or via metabolic activities within the plant tissue), phytovolatilization (uptake of contaminants by the plant root system and their subsequent release in a gaseous form), and phytoextraction (accumulation of contaminants in the aboveground parts of plants) (Greipsson 2011). The dominant phytoremediation mechanism responsible for the results discussed in this paper is phytoextraction. This mode of phytoremediation requires regular harvesting of biomass to decrease the contaminant concentration in the soil. In the investigations, both continuous and induced phytoextraction was practiced (continuous phytoextraction refers to the use of fast-growing plants, such as giant miscanthus to treat the soil without soil/process modifications; induced phytoremediation involves the use of, e.g., chemicals which aim at increasing bioavailability of pollutants for plants; in this paper, bioavailability of pollutants was enhanced by using a soil improver).

Plants useful for phytoremediation applications must meet some requirements, such as ease of planting and cultivation, good adaptability, fast growth, high biomass yield, and high tolerance to contaminants (Bian et al. 2020; Xiao et al. 2021; Yang and Shen 2020). Nowadays, special attention has been paid to the potential of energy crops for phytoremediation processes, which allows the exploitation of the synergistic effect of soil treatment and energy production (Grifoni et al. 2021; Prabha et al. 2021). The cultivation of energy plants is considered a promising solution for the future, being a carbon–neutral, clean, and eco-friendly renewable energy source. The biomass obtained can be easily converted to bio-solid, bio-liquid, and bio-gaseous fuels using thermochemical or biological methods (Grzegórska et al. 2020).

*Miscanthus* × *giganteus* J.M.Greef & Deuter, known as giant miscanthus, is an energetic plant characterized by ecological adaptability and large biomass production. Furthermore, having good tolerance to nutrient deficiency, a wide temperature range, and a high accumulation of heavy metals and other pollutants, it is suitable for soil treatment (Dubis et al. 2020; Magenau et al. 2021; Voća et al. 2021). This grass is also easily spread vegetatively by cuttings of rhizomes (Pidlisnyuk et al. 2020a). Recent studies carried out by Pidlisnyuk et al. (Pidlisnyuk et al. 2020b) evaluated the dynamic of concentration of Ti, Mn, Fe, Cu, Zn, As, Sr, and Mo during the application of giant miscanthus. Despite the soil contamination, proper development of the plant with sufficient biomass production was observed. However, the volume of the plants decreased with a higher level of soil contamination. Furthermore, authors pointed out the differences in metal(loid)s’ behavior in the plants’ organs depending on vegetation year, the concentration of elements in the substrate, and their nature. Meanwhile, Andrejić et al. (Andrejić et al. 2019) evaluated the effects of mineral NPK fertilization on metal accumulation, physiological parameters, and growth of giant miscanthus cultivated on flotation tailings. Obtained results showed that giant miscanthus acts as an excluder of Cu, Zn, and especially Pb. The authors also noticed that fertilization enhanced metal uptake by plant roots, but had no effect on their translocation to leaves. Voća et al. (Voća et al. 2021) investigated the effect of the addition of municipal sewage sludge on the biomass yield and the energy properties of giant miscanthus. The authors concluded that the use of municipal sewage sludge did not increase the content of heavy metals in the soil and plant biomass, but led to improved quality of the soil properties.

The effects of applying various amendments such as chemical (e.g., citric acid, EDTA, EGTA, SDS) and organic amendments (e.g., composts, sewage sludge, humic substances, biochar) to agricultural crops have been widely studied in the literature. However, plenty of these studies focused on the analyses regarding organic and inorganic components, nutrients, and granulometric composition. Such physico-chemical analysis cannot evaluate the effects of amendments on living organisms. It is, among others, due to the response of living organisms not only to single compounds but a possibility of synergistic or antagonistic interactions between components of mixtures of compounds that constitute amendments. Therefore, biotests (or bioassays) are advised to be performed prior to agricultural or environmental application of soil amendments (Gondek and Mierzwa-Hersztek 2017; Antonkiewicz et al. 2019a). The application of bioassay is an efficient and useful method for predicting environmental risk to ecosystems. By conducting biological tests, we can assess the scale, intensity, and effects of pollution, as well as the ability of the site/soil to self-repair, self-cleaning, or select, for example, a plant species with phytoremediation potential. The ecotoxicological analysis may be incorporated as a so-called parallel monitoring system to validate the efficiency of phytoremediation. The combination of chemical methods with biological tests could provide a more complex tool to establish the originality of the toxicant and its potential risk to human health. Bioassays are particularly important in field conditions as a simple and cost-effective method which let to determine not only the effects of pollution but also the progress of phytoremediation (Paisio et al. 2021; Bharti and Banerjee 2013; Varun et al. 2011). One of the advantages of biotests is determining the cumulative toxicity of mixtures of both known and unknown chemicals present in a matrix. Thus, bioassays can be used in the evaluation of the effects and interactions of all compounds present in soil on living organisms. In order to obtain a high accuracy of evaluation with respect to soil and sludge ecotoxicity tests, a model test organism should represent various levels of the trophic chain and be able to respond to chemical substances (Oleszczuk and Hollert 2011; Baran and Tarnawski 2013). The utilization of such biotests enables the identification of potential threats related to the valorization of soil, sludge, or soil amendments.

In this regard, this study involves the application of giant miscanthus for the remediation of soils contaminated with metals. The novelty of the presented research includes a field experiment performed on the territory of a Municipal Waste Rendering Plant. A literature study reveals that phytoremediation actions have previously been carried out on landfills or leachate from landfills (Abbas et al. 2019; Bhagwat et al. 2018; Johar et al. 2017; Jones et al. 2006; Kim and Owens 2010; Nagendran et al. 2006; Pathak et al. 2012). However, the main aim of our study was related to comparing the efficiency of the phytoremediation process on various substrates. One of the directions was the evaluation of the phytoremediation efficiency by improving the land quality with the use of waste processing products. These products can be considered as a soil improver, and this is the case with the Municipal Waste Rendering Plant in Gdańsk. The soil improver used in the experiment contained a high content of organic matter, which is necessary for the natural management of post-industrial areas with unfavorable physical and chemical properties. The use of a soil improver as an organic fertilizer may improve the physicochemical properties of chemically contaminated soil, and the organic matter contained in it, in addition to water retention, leads to limits the mobility of heavy metals. On the other hand, we compare the effect of the addition of highly contaminated post-industrial soil on plant growth and phytoremediation effectiveness. Such application of the post-industrial soil, highly contaminated with metals, possibly cleaned up during the phytoremediation process, in the future will allow it to be used as a substrate for the creation of rehabilitation layers in a municipal landfill.

## Materials and methods

Research on the treatment of the contaminated soil using giant miscanthus was carried out in an area of the Municipal Waste Rendering Plant in Gdańsk — Szadółki, located in northern Poland (54° 19′ 12.107″ N, 18° 32′ 23.62″ E).

### Soil and SK-8

The soil in which the experiment was established was classified as clay loam (CL) (Marcinek and Komisarek 2011; Soil Survey Staff 2014). The selected physicochemical properties of the Municipal Waste Rendering Plant soil, soil improver (SK-8), and highly contaminated post-industrial soil are presented in Table 1. SK-8 is a product of the Municipal Waste Rendering Plant in Gdańsk. The fertilizer is made in the process of composting biodegradable waste that is accessible to the disposal facility. SK-8 can be used to improve the physical and chemical properties of all types of soil, including the cultivation of ornamental plants and lawns, as a component of the cultivation of potted plants or balconies and terrace plants, as well as in the rehabilitation of degraded areas. It is especially recommended for soils with a low humus content (“Soil improver Certificate,” 2017).

**Table 1:** Selected physicochemical properties of the soils and the SK-8

Parameter	Unit	Municipal waste rendering plant soil*	SK-8	Highly contaminated post-industrial soil
Dry mass	%	-	58.25 ± *3.04*	-
pH_H2O_	-	5.52 ± 0.10^**^	7.34 ± *0.06*	5.82 ± *0.10*
pH_KCl_	-	4.80 ± *0.14*	7.23 ± *0.13*	5.05 ± *0.08*
The sum of exchangeable cations	mmol( +)∙kg^−1^	143.1 ± *8.1*	-	184.7 ± *8.8*
electrical conductivity	mS∙cm^−1^	4.53 ± *0.10*	1.74 ± *0.08*	5.10 ± *0.14*
Organic carbon	g∙kg^−1^ d.m	8.77 ± *0.33*	221.1 ± *9.7*	6.49 ± *0.67*
Available macronutrients				
Phosphorus (P)	mg∙kg^−1^ d.m	20.28 ± *1.13*	-	13.13 ± *1.39*
Potassium (K)		79.88 ± *1.51*	-	55.36 ± *11.90*
Magnesium (Mg)		37.77 ± *1.90*	-	22.43 ± *2.68*
Total macronutrients				
Nitrogen (N)	g∙kg^−1^ d.m	0.39 ± *0.02*	5.30 ± *0.19*	1.15 ± *0.03*
Phosphorus (P)		0.34 ± *0.03*	1.42 ± *0.06*	0.39 ± *0.03*
Potassium (K)		0.94 ± *0.05*	4.82 ± *0.25*	1.01 ± *0.29*
Microelements and heavy metals				
Aluminum (Al)	mg∙kg^−1^ d.m	9950.0 ± *625.9*	3538.0 ± *606.6*	8422.0 ± *905.5*
Iron (Fe)		12,492.0 ± *743.4*	1457.7 ± *84.9*	11,315.0 ± *888.5*
Zinc (Zn)		513.3 ± *14.0*	133.8 ± *14.2*	487.3 ± *26.8*
Cadmium (Cd)		4.6 ± *0.3*	1.8 ± *0.3*	6.1 ± *0.2*
Chrome (Cr)		73.0 ± *10.4*	54.2 ± *8.5*	71.1 ± *5.2*
Cobalt (Co)		10.7 ± *1.5*	5.2 ± *1.1*	9.3 ± *0.9*
Copper (Cu)		496.7 ± *13.1*	85.2 ± *7.0*	254.6 ± *18.0*
Lead (Pb)		284.7 ± *12.3*	115.3 ± *8.7*	439.0 ± *11.6*
Manganese (Mn)		284.8 ± *8.6*	58.4 ± *4.0*	247.4 ± *7.2*
Nickel (Ni)		15.4 ± *0.8*	29.2 ± *3.0*	17.3 ± *0.9*

d.m. - dry matter

^*^Sandy loam

^**^SD — standard deviation

### The scheme and conditions of the research

In 2020, a one-factor field experiment was established in a randomized block design with three repetitions. The aboveground parts of plants were collected in 2021 after 1 year of plants vegetation, when the average annual rainfall was 531 mm, while the average annual temperature was about 11 °C. The plot area was 51.75 m^2^ (11.5 × 4.5 m) with a 0.5-m spacing between the plots. The experimental design included 4 objects: 1 — soil of Municipal Waste Rendering Plant in Gdańsk, 2 — soil of Municipal Waste Rendering Plant mixed with 200 Mg‧ha^−1^ of SK-8; 3 — soil of Municipal Waste Rendering Plant mixed with 400 Mg ha^−1^ of SK-8, and 4 — soil of Municipal Waste Rendering Plant mixed with 100 Mg ha^−1^ of highly contaminated post-industrial soil (as shown in Table 2). SK-8 and the post-industrial soil were applied to the soil surface once in spring 2020, then mixed with it for 2 weeks before planting giant miscanthus seedlings. Initially, giant miscanthus seedlings were sprinkled with water as the substrate dried to a depth of 10 cm. In this field experiment, no mineral fertilization (NPK) was applied, assuming that SK-8 was used as a source of nutrients for plants.

**Table 2:** Combinations and additives used in the field experiment

Object no.	Material/combination	Dose of additive (Mgꞏha^−1^)
1	Municipal Waste Rendering Plant soil	0
2	Municipal Waste Rendering Plant soil mixed with single dose of SK-8	200
3	Municipal Waste Rendering Plant soil mixed with double dose of SK-8	400
4	Municipal Waste Rendering Plant soil mixed with highly contaminated post-industrial soil	100

### Plant characterization

In this research, giant miscanthus plants derived from the Nursery of Ornamental Plants Paweł Pesta (Lubichowska St. 9, 83–200 Starogard Gdanski, Poland) have been used. According to the European Union Plant Passport, the RUOP registration code is PL-22/13/77, the company traceability code is PW 6/01/2020/12736, and the country of origin is Poland.

### Determination of the dry matter yield and the content of elements in the soil and plants

#### Plant material

The investigated samples of giant miscanthus (aboveground parts of plants) were collected from all of the field experiments (objects 1–4) in 2021 after 1 year of vegetation. The harvested fresh plant material was dried in a laboratory dryer (SLN 32 SMART, POL-EKO APARATURA, Wodzisław Śląski, Poland) with forced air circulation at 105 °C to a constant weight. The dried plant material was weighed, and the yield of plants from each plot was determined. For the evaluation of the yield, the obtained yield per plot was converted to an area of 1 ha. The dried plant samples were ground on an Ultra Centrifugal Mill (ZM 200, RETSCH, Haan, Germany). During grinding, sieves with a 4- and 1-mm-diameter mesh were used. The material after grinding was stored in sealed containers at room temperature.

The mineralization process of the plants was performed with the use of the Magnum II microwave mineralization system (Ertec, Wroclaw, Poland). The process was carried out in 7 ml of Suprapur grade 65% HNO_3_ solution (Avantor Performance Materials S.A., Gliwice, Poland). The masses of the mineralized plant samples were 0.3 ± 0.005 g. The process was performed in 3 cycles to prevent rapid pressure increase. Cycle conditions are summarized in Table 3.

**Table 3:** Microwave mineralization cycle conditions

Cycle	Time (min)	Pressure (bar)	Temperature (^°^C)
I	5	23–25	140–150
II	5	30–35	240–250
III	20	43–46	295–300

Selected element (Al, Fe, Mn, Co, Zn, Cd, Cr, Cu, Ni, and Pb) determination was performed with the use of a microwave plasma-atomic emission spectrometer 4210 MP-AES (Agilent Technologies Inc., Santa Clara, CA, USA). The concentration measurements of the selected elements were performed with 4 repetitions and in 3 separate procedures with different wavelengths. The procedure was performed to check and reduce the possible impact of spectral interferences. Uncertainties were presented as standard uncertainty from all 4–12 measurements considering the calibration uncertainty.

#### Soil materials

The determination of basic physicochemical properties of the soils were performed in the top layer of Municipal Waste Rendering Plant soil (0–25 cm), in SK-8, and the highly contaminated post-industrial soil (as shown in Table 1). The following generally accepted methods used in accredited laboratories were applied: dry mass using the drying method, soil pH–pH in H_2_O and 1 mol ∙ dm^−3^ KCl (soil: solution = 1:2.5) determined potentiometrically (PN-ISO-10390–1997 1997), electrical conductivity with a conductometer, organic carbon using the Tiurin method, total nitrogen using the Kjeldahl method (Jones and Case 1990; Ostrowska et al. 1991). The content of available forms of phosphorus and potassium in the soil was determined by the Egner-Riehm method according to the PN-R-04023: 1996 and PN-R-04022: 1996 standards (PN-R-04022–1996 1996; PN-R-04023–1996 1996), respectively, and the available magnesium content according to the PN-R-04020: 1994 standard (PN-R-04020–1994 1994). The sum of the exchangeable cations called as cation exchange capacity was measured by summation method determined by the summing exchangeable Ca, Mg, Na, K, and Al. The content of metal elements (Al, Fe, Zn, Cd, Cr, Co, Cu, Pb, Mn, and Ni) in soil samples was determined according to the method described in “Plant material.”

#### Ecotoxicological analysis

Ecotoxicity of the soil was evaluated using 2 biotests: Phytotoxkit and Ostracodtoxkit. The Phytotoxkit was performed for the three plants: *Lepidium sativum* L., *Sinapis alba* L., and *Sorghum saccharatum* (L.) Moench. The determined parameters were inhibition of seed germination and inhibition of root length after their 3-day incubation with soil samples. The Ostracodtoxkit biotest measured the mortality and inhibition of growth of *Heterocypris incongruens* after a 6-day exposure of the crustacean to samples. Both biotests were performed according to the procedure created by the producer (Ostracodtoxkit 2001; Phytotoxkit 2004). The results obtained were expressed as a test reaction percentage effect (PE). The toxicity of the samples was assessed according to the following criteria: class I (PE ≤ 20% no significant toxic effect) − no acute hazard; class II (20% < PE ≤ 50% significant toxic effect, low toxic sample) − low acute hazard; class III (50% < PE < 100% significant toxic effect, toxic sample) − acute hazard; class IV (PE = 100% single test) − high acute hazard; class V (PE = 100% all tests) − very high acute hazard (Persoone et al. 2003).

#### Quality control of the analysis

The measurements for each of the analyzed samples were carried out at three repetitions. The accuracy of the analytical methods was verified on the basis of certified reference materials and standard solutions: CRM IAEA/V—10 Hay (International Atomic Energy Agence), CRM—CD281—Rey Grass (Institute for Reference Materials and Measurements), and CRM023-050—Trace Metals—Sandy Loam 7 (RT Corporation).

### Calculations

The following parameters were adopted on the basis of which phytorecovery of elements from chemically contaminated soil was determined:

1. Dry matter yield (*Y*);
2. Content of Al, Fe, Zn, Cr, Co, Cu, Pb, Mn, and Ni in the yield;
3. The uptake of elements (*U*) was calculated as the product of the dry matter yield (*Y*) and the content of the component (*X*), according to the formula: *U* = *Y* ∙ *X*;
4. The balance of elements was calculated from the difference between the amount of elements introduced with the single/double dose of SK-8 and highly contaminated post-industrial soil, and the amount of components taken with the crop yield;
5. Phytorecovery of elements was presented as the percentage of uptake of these components in relation to the amount added to the soil with additives.

### Statistical analysis

The statistical analysis of the research results was performed using the Microsoft Office Excel 2013 spreadsheet and the Statistica 13 PL software. Statistical evaluation of the variability of the results was performed using a one-way analysis of variance. The significance of the differences between the mean values was verified based on the *t*-Tukey test at the significance level *α*  ≤ 0.05. For selected relationships (parameters), the value of Pearson’s linear correlation coefficient (*r*) was calculated, with the significance level of *α*  ≤ 0.05.

## Results and discussion

### Physicochemical properties of soil and SK-8

The soil in which the experiment was established was acidic, and SK-8 was neutral (Ostrowska et al. 1991). The content of the sum of exchangeable cations in the soil of the field experiment was comparable to the heavy soils of southern Poland (Mierzwa-Hersztek et al. 2016). Electrical conductivity is taken as a measure of soil salinity. The impact of high values of soil salinity on plants is manifested mainly in difficulties in water uptake, which is important in the phytoextraction of pollutants from post-industrial soils, including landfills. The range of electrical conductivity at the level of 2–4 mS∙cm^−1^ is considered to reduce the yield of more sensitive plants (Hogg et al. 2002). The electrical conductivity values measured in the soil of the field experiment showed high values, and even higher values were recorded for the post-industrial soil. Despite the high values of electrical conductivity in the studied soils, the cultivated giant miscanthus did not show any signs of sensitivity to the salinity of the soil. The marked feature of giant miscanthus can also be used in the remediation of highly saline soils (Kamiński et al. 2021). The SK-8 used in the experiment was characterized by an average salinity and comparable to that of typical urban composts (Kujawska et al. 2016). The organic carbon content in the Municipal Waste Rendering Plant soil and in the highly contaminated post-industrial soil was at the average level for soils in Poland (Mierzwa-Hersztek et al. 2016). Based on the organic carbon content, the SK-8 used can be classified as organic fertilizers (MARD-Regulation 2008). The content of available phosphorus, potassium, and magnesium in the studied soil of the Municipal Waste Rendering Plant soil and highly contaminated post-industrial soil was at a very low level (Ostrowska et al. 1991). The content of basic macronutrients in the Municipal Waste Rendering Plant soil and highly contaminated post-industrial soil were within the range for arable soils in Poland (Tkaczyk et al. 2017). These soils were characterized by an average content of Al, Mn, Fe, and Co in the soils of Poland (Korzeniowska and Krąż 2020). The content of the microelements mentioned above in the used SK-8 was comparable to the composition of composts from large urban agglomerations (Gondek et al. 2014).

### Assessment of heavy metals content in soils and SK-8 according to applicable standards

According to the current regulation, the analyzed land was included in group IV, as post-industrial areas (MARD-Regulation 2016). On post-industrial land, the permissible heavy metal content is much higher than on arable land, where biomass is produced for consumption. The heavy metal content in the soils, in which the field experiment was established, was below the permitted limits for post-industrial areas (Regulation EU 2019/1009). Furthermore according to the current Regulation MARD 2008 (MARD-Regulation 2008), the content of heavy metals in soils and in SK-8 did not exceed the permissible values for their agricultural and recultivation use (referred in Table 4).

**Table 4:** Quality standards for soil and composts approved for use in the environment (mg∙kg d.m.)

Standards for	Heavy metal					
	Cr	Ni	Cu	Zn	Cd	Pb
Soil^*^	1000	500	600	2000	15	600
Compost^**^	100	60	-	-	5	140

^*^Regulation 2016 (MARD-Regulation, 2016)

^**^Regulation MARD 2008 (MARD-Regulation, 2008)

The main source of elements for giant miscanthus was the Municipal Waste Rendering Plant soil and the highly contaminated post-industrial soil. The content of Al, Fe, Mn, Co, Zn, Cd, Cr, Cu, and Pb found in these soils was much higher than in SK-8. The exception was nickel, the highest content of which was found in SK-8 and the lowest in the soil of the Municipal Waste Rendering Plant soil. Previous studies (Kicińska and Wikar 2021; Pavel et al. 2014) confirm that post-industrial lands are characterized by high chemical pollution resulting from industrial activities. Such soil should be remediated, including phytoextraction, or stabilized by precipitating pollutants into less mobile forms (Antonkiewicz et al. 2019b; Liu et al. 2018, 2021).

### Yield of plants

**Fig. 1:**
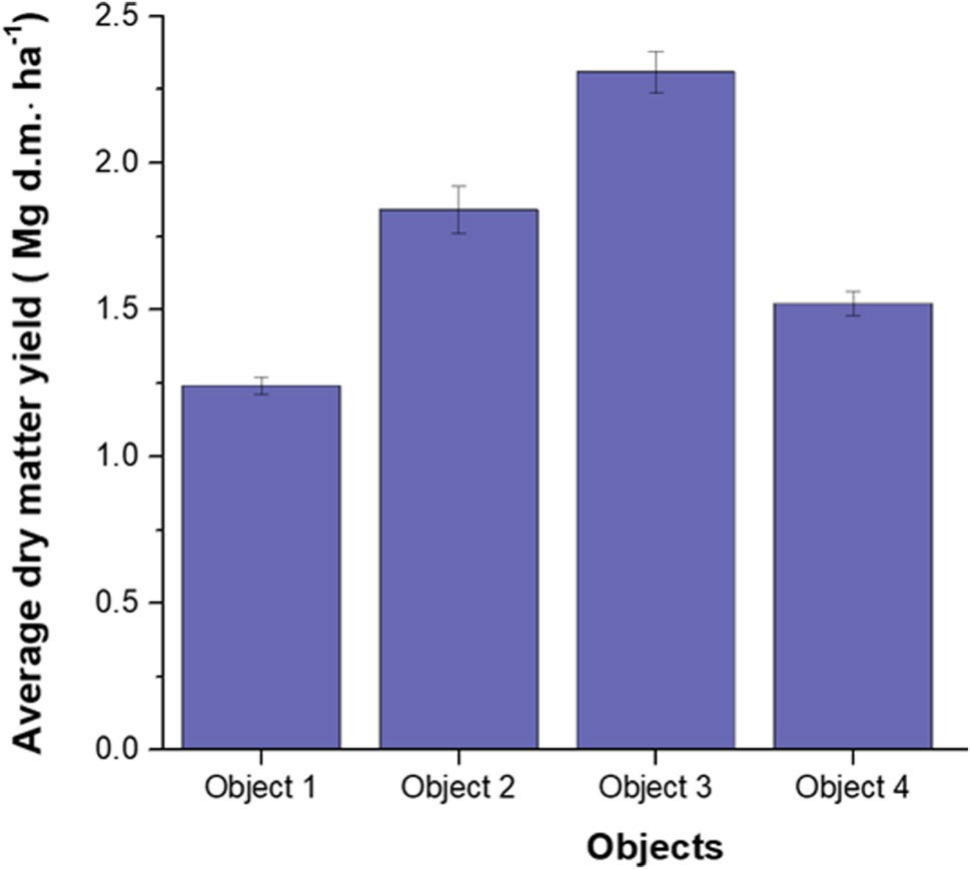
Average giant miscanthus dry matter yield in 2021 for objects 1–4

The average dry matter yield of giant miscanthus cultivated on objects 1–4 ranged from 1.24 to 2.31 Mg d.m.∙ha^−1^ (as shown in Fig. 1). The lowest dry matter yield was obtained in the soil without additives (object 1) while increasing doses of SK-8 (objects 2 and 3) significantly increased the yield of the plants. The research carried out shows that the highest yield-generating effect was obtained in object 3 where 400 Mg‧ha^−1^ of SK-8 was applied. The increase in yield in this facility was more than 85% compared to object 1. Meanwhile, in object 4 an increase in yield of over 22% compared to object 1 was recorded.

The analysis of the linear correlation (*r*) by Pearson shows a close relationship between the load of the introduced elements with SK-8 and highly contaminated post-industrial soil and the giant miscanthus yield (*r* = 0.606–0.981). The research shows that the calculated correlation relationships show a significant impact of the elements load from Municipal Waste Rendering Plant soil, SK-8, and highly contaminated post-industrial soil on the yield of the tested plant.

### The content of microelements and heavy metals in giant miscanthus

As shown in Table 5, significant differences was observed for metal content in giant miscanthus for various objects. Among the elements analyzed, the highest content of Zn, Cr, Cu, and Mn was found in giant miscanthus grown in object 1. Importantly, the application of a single dose of SK-8 (object 2) increased the content of Al, Fe, Pb, Ni, and Cd compared to object 1. Giant miscanthus grown on a double dose of SK-8 (object 3) contained more Al and Pb compared to object 1. The highly contaminated post-industrial soil (object 4) was a potential source of cadmium for the tested plants. The cadmium content in the plants biomass obtained from object 4 was the highest compared to all objects.

**Table 5:** Average metal content in giant miscanthus (mg ꞏ kg^−1^ d.m.)

Object	Al	Fe	Mn	Co	Cu
1	74.9 ± *3.4*	227.0 ± *12.9*	25.2 ± *1.4*	0.61 ± *0.05*	104.2 ± *2.9*
2	323.8 ± *13.6*	562.2 ± *22.0*	21.8 ± *0.2*	0.63 ± *0.03*	9.3 ± *0.2*
3	118.3 ± *5.6*	209.3 ± *3.0*	10.6 ± *0.6*	0.64 ± *0.01*	9.4 ± *0.1*
4	106.6 ± *4.7*	219.2 ± *3.1*	22.0 ± *0.1*	0.61 ± *0.02*	5.8 ± *0.3*
Mean value	155.9	304.4	19.9	0.62	32.2
LSD_0.05_	14.9	24.3	1.5	0.06	2.7
CV%	72.8	56.5	32.1	2.45	149.2
Object	Zn	Cd	Cr	Pb	Ni
1	131.4 ± *3.1*	0.12 ± *0.02*	12.6 ± *0.9*	2.9 ± *0.2*	7.0 ± *0.1*
2	50.3 ± *1.9*	0.16 ± *0.02*	5.6 ± *0.2*	6.3 ± *0.5*	11.6 ± *0.2*
3	52.5 ± *3.4*	0.12 ± *0.01*	3.2 ± *0.1*	6.7 ± *0.1*	2.6 ± *0.1*
4	58.2 ± *4.0*	0.17 ± *0.03*	6.5 ± *0.2*	3.8 ± *0.2*	6.8 ± *0.2*
Mean value	73.1	0.14	7.0	5.0	7.0
LSD_0.05_	6.0	0.04	0.8	0.5	0.4
CV%	53.4	17.51	57.3	37.4	52.2

^*^CV — variability coefficient

^**^LSD — least significant difference

The correlation analysis showed a negative relationship between the load of the introduced elements with SK-8 and highly contaminated post-industrial soil and the content of Mn, Cu, and Zn in plant biomass (*r* =  − 0.594 to − 0.949). A negative correlation was also found between the plant yield and the content of Mn, Cu, and Zn in biomass (*r* =  − 0.680 to − 0.909). The aforementioned correlation relations prove a significant influence of additives on the quality of biomass assessed in terms of phytoextraction.

### Uptake of microelements by plants

The amount of elements taken from the substrates (Table 6) depended on the dry matter yield and the content of a given element in the yield (Fig. 1; Table 5). In the Municipal Waste Rendering Plant soil (object 1), where no additives were used, it was found that giant miscanthus took up the most Zn, Cr, and Cu compared to the other objects. The highest uptake of the elements mentioned above is also explained by the highest content of these elements in the soil where giant miscanthus was grown. The research shows that the giant miscanthus used can be dedicated to phytoextraction of the elements mentioned above from barren, chemically contaminated soil, without fertilization with organic matter (Pidlisnyuk et al. 2021). Research by other authors (Lee et al. 2022) confirms that giant miscanthus is suitable for the phytoextraction of chemical pollutants from post-industrial soils.

**Table 6:** Uptake of the metal elements by giant miscanthus (g ha^−1^)

Object	Al	Fe	Mn	Co	Cu
1	92.5 ± *1.8*	280.5 ± *11.7*	31.1 ± *0.8*	0.75 ± *0.05*	128.8 ± *0.5*
2	596.3 ± *30.5*	1036.3 ± *83.0*	40.3 ± *2.3*	1.16 ± *0.10*	17.1 ± *0.8*
3	273.1 ± *1.5*	482.6 ± *24.1*	24.5 ± *2.3*	1.48 ± *0.04*	21.7 ± *0.7*
4	161.8 ± *1.5*	333.0 ± *16.8*	33.4 ± *2.1*	0.93 ± *0.08*	8.9 ± *1.0*
Mean value	280.9	533.1	32.3	1.08	44.1
LSD_0.05_	38.9	83.6	3.8	0.14	1.5
CV%	79.4	64.9	20.1	28.97	128.5
Object	Zn	Cd	Cr	Pb	Ni
1	162.5 ± *1.3*	0.15 ± *0.02*	15.5 ± *0.6*	3.6 ± *0.3*	8.7 ± *0.4*
2	92.6 ± *5.2*	0.30 ± *0.04*	10.3 ± *0.8*	11.7 ± *1.2*	21.4 ± *1.7*
3	121.1 ± *9.0*	0.28 ± *0.03*	7.4 ± *0.4*	15.5 ± *0.9*	6.1 ± *0.4*
4	88.3 ± *1.0*	0.25 ± *0.06*	9.9 ± *0.9*	5.8 ± *0.1*	10.3 ± *1.0*
Mean value	116.1	0.25	10.8	9.2	11.6
LSD_0.05_	9.9	0.08	1.4	1.4	1.9
CV%	29.4	26.46	31.7	59.1	58.0

^*^CV — variability coefficient

^**^LSD — least significant difference

The application of a single dose of SK-8 (object 2) increased the uptake of Al, Fe, Co, Pb, Mn, Ni, and Cd by plants compared to object 1. Doubling the dose of SK-8 (object 3) also increased the uptake of Al, Fe, Co, Pb, and Cd compared to object 1. Our own research shows that the application of SK-8 “activated” the abovementioned elements, which became more accessible to giant miscanthus. The application of organic matter to sterile chemically contaminated soils, among others, changes the soil reaction, and thus increases the bioavailability of elements for the root system of remediation plants (Pavel et al. 2014; Peña et al. 2020). In the case of Cr, Zn, Cu, Mn, and Ni, it is observed that the addition of organic matter, especially in an SK-8 double dose, reduced the amount of the abovementioned elements compared to object 1 (Table 6). The reduction in the amount of the collected elements with the higher dose of SK-8 despite higher plant biomass yield may resulted, among others, from the strong binding of heavy metals by the functional groups of the organic matter in SK-8 (Asada et al. 2010; Liu et al. 2021.) leading to the reduction in the bioavailability of metals in soil and therefore plant accumulation.

The use of highly contaminated post-industrial soil (object 4) increased the uptake of Al, Fe, Co, Pb, Mn, Ni, and Cd by plants compared to object 1. The collection of the abovementioned elements is also explained by the high content of these elements in the used soil. The research shows that giant miscanthus can be used for phytoextraction of elements from post-industrial soils as a good and efficient phytoextractor, which is also confirmed by other studies (Pidlisnyuk et al. 2021).

Statistical analysis showed a close relationship between the amount of introduced elements with SK-8 and highly contaminated post-industrial soil and the uptake of Co, Cu, and Zn by giant miscanthus (*r* = 0.590–0.969). A positive correlation was also found between the yield and Co uptake by giant miscanthus (*r* = 0.991), while there was a negative correlation between the yield and Cu uptake by giant miscanthus (*r* =  − 0632). The field experiment showed significant relationships between the contents of Al, Fe, Mn, Co, Cu, Zn, Cd, Cr, Pb, and Ni and the uptake of these elements by giant miscanthus (*r* = 0.577–0.997). The above correlations indicate a significant influence of the experimental factors on the intensity of the process of uptake of elements by giant miscanthus in the aspect of phytoextraction and phytonutrition of elements from the substrate.

### Simplified balance and phytorecovery of elements

The study attempts to evaluate the suitability of giant miscanthus for the phytoremediation of elements from chemically contaminated substrates. The application of large amounts of SK-8 (200 and 400 Mg‧ha^−1^) to soil on a rendering plant territory to create a reclamation layer probably contributed to the immobilization of toxic elements, and thus reduced the possibility of including them in the biological cycle. Understanding the circulation of elements in the soil-waste-plant system through a simplified balance allows for assessing the effectiveness of the phyto-cleaning treatment of waste substrates from contaminating elements (Burger et al. 2020). On the other hand, the percentage of phytonutrients presented will indicate which of them is recovered most effectively from the substrate. In the balance calculations, the difference between the amount of elements introduced with the doses of SK-8 and post-industrial soil and the uptake of these components by plants was assumed (Table 7). In the soil without additives (object 1), the balance was negative. The negative value of the balance resulted from the adopted calculation method, in which the inflow of elements from external sources was not taken into account. External sources of elements include the content of assimilable forms of these components in the soil, the mineralization of organic matter or precipitation, and the wet deposition of dust-containing pollutants (Ustak and Munoz 2018; Wieczorek et al. 2021). The research shows that the use of SK-8 and highly contaminated post-industrial soil (objects 2–4) resulted in a positive balance for the elements analyzed in giant miscanthus. The positive value of the balance of elements resulted mainly from a greater amount of these components with additives than their total uptake by plants.

**Table 7:** Simplified balance and phytorecovery of elements

Object	Introduced	Uptake	Balance	Recovery	Introduced	Uptake	Balance	Recovery
	(g ∙ ha^−1^)			(%)	(g ∙ ha^−1^)			(%)
	Al				Fe			
1	0	93	− 93	-	0	281	− 281	-
2	707,733	596	707,137	0.013	291,533	1036	290,497	0.096
3	1,415,467	273	1,415,194	0.019	583,067	483	582,584	0.083
4	842,200	162	842,038	0.019	113,1567	333	1,131,234	0.029
	Zn				Cr			
1	0	162	− 162	-	0	16	− 16	-
2	26,753	93	26,661	0.607	10,847	10	10,836	0.143
3	53,507	121	53,386	0.226	21,693	7	21,686	0.034
4	48,730	88	48,642	0.181	7113	10	7103	0.139
	Co				Cu			
1	0	0.7	− 0.7	-	0	129	− 129	-
2	1047	1.2	1046	0.072	17,047	17	17,030	0.755
3	2093	1.5	2092	0.070	34,093	22	34,072	0.064
4	930	0.9	929	0.100	25,460	9	25,451	0.035
	Pb				Mn			
1	0	4	− 4	-	0	31	− 31	-
2	23,067	12	23,055	0.016	11,680	40	11,640	0.266
3	46,133	16	46,118	0.034	23,360	24	23,336	0.105
4	43,900	6	43,894	0.013	24,737	33	24,703	0.135
	Ni				Cd			
1	0	9	− 9	-	0	0.15	− 0.15	-
2	5840	21	5819	0.149	356	0.30	356	0.043
3	11,680	6	11,674	0.052	712	0.28	712	0.039
4	1730	10	1720	0.594	608	0.25	607	0.042

Among the elements analyzed, it was found that for soil with a double dose of SK-8 (object 3), the most introduced was Al, followed by Fe, Zn, Pb, Cu, Mn, Cr, Ni, Co, and Cd. On the other hand, the greatest uptake of elements, depending on the object, concerned Fe, in the order Al, Mn, Zn, Cu, Mn, Ni, Cr, Pb, and Cd. In summary, it was found that the largest balance difference was recorded in the object, where the highest dose of SK-8 (400 Mg‧ha^−1^) was applied.

Based on a field experiment, the estimated recovery of elements (Table 7) depends on the amount of elements introduced into the soil and the amount of their uptake by giant miscanthus. The phytorecovery of elements from the ground was very small and did not exceed 1% of the amount introduced into the soil, probably due to the short growing season of giant miscanthus and the use of large doses of SK-8 and post-industrial soil.

Among the elements analyzed, depending on the object, it was found that giant miscanthus recovered the highest percentage of Cu (0.755%), followed by Zn, Ni, Mn, Cr, Co, Fe, Cd, Pb, and the least amount of Al (0.019%). The highest percentages of Cu, Fe, Cr, Mn, and Cd phytonutrition were found from a soil with a single dose of SK-8 (object 2), Pb and Al from a soil with double dose of SK-8 (object 3), and Co and Ni from the soil mixed with highly contaminated post-industrial soil (object 4). The above series shows that giant miscanthus recovered Cu in the highest percentage, while Al recovered in the lowest percentage, which was mainly due to the amount of components introduced with SK-8.

The correlation analysis showed strong relationships between the amount of introduced Fe, Mn, Cu, Zn, Cr and Ni with SK-8 and post-industrial soil, and the phytonutrition of these elements. The above relationships were negatively correlated, which indicates that the more elements were introduced into the soil, the lower the phytorecovery was. This dependence is of great importance in the rate of the purification process; thus, the higher the level of soil contamination, the more time the soil cleanup process with plants will take, which is also confirmed by other studies (Shen et al. 2021).

### Ecotoxicity of soils

Phytotoxkit tests find application in the evaluation of ecotoxicity of various materials, including sludges, sediments, soils, or waste (Antonkiewicz et al. 2019a; Kopeć et al. 2013; Urbaniak et al. 2020). In this work, phytotoxicity of investigated soils was assessed based on the regression of both germination and root growth of plants (Table 8). It was noted that the inhibition of germination was about 0 to 5% while the inhibition of root growth was in the range between − 19 and 16%. The results of bioassay suggest that *L. sativum* seems to be the most sensitive compared to *S. alba* and *S. saccharatum*. However, it is worth of note that for selected experimental objects, a test plant growth stimulation was observed and both evaluated parameters (i.e., inhibition of germination and root growth) confirmed lack of toxicity of investigated soils towards test plants. All investigated soil samples were nontoxic to Phytotoxkit test plants (PE ≤ 20%).

**Table 8:** Results of various ecotoxicity tests

Biotests		Object 1	Object 2	Object 3	Object 4	Mean value
		PE%				
*S. alba*	GI	0	0	1 ± *2*	3 ± *2*	1
	RI	− 18 ± *7*	4 ± *6*	10 ± *5*	− 19 ± *6*	− 6
*L. sativum*	GI	4 ± *5*	2 ± *4*	0	3 ± *4*	2
	RI	10 ± *12*	13 ± *5*	16 ± *11*	− 2 ± *16*	9
*S. saccharatum*	GI	4 ± *5*	2 ± *4*	4 ± *2*	6 ± *9*	4
	RI	− 6 ± *10*	− 5 ± *11*	2 ± *9*	− 16 ± *11*	− 6
*H. incongruens*	M	0	0	0	0	0
	IG	37 ± *9*	45 ± *9*	16 ± *10*	20 ± *8*	30

*GI* germination inhibition, *RI* roots growth inhibition, *M* mortality, *IG* growth inhibition

The Ostracodtoxkit test is used in the evaluation of toxicity of solid media via a direct contact between a test organism (here *Heterocypris incongruens*) and a medium. It was noted (Table 8) that the growth inhibition of *H. incongruens* was between 16 and 45%, depending on the soil type. The highest toxic reactions of *H. incongruens* were observed for object 2, while the lowest were noted for object 3. Objects 1 and 2 were classified as low toxic and objects 3 and 4 as non-toxic with respect to growth inhibition of *H. incongruens*.

It is worth noting that germination and root growth are the critical phases of development for plants sensitive to soil quality and pollution. Interestingly, results presented in this paper show that the investigated soils exhibited higher ecotoxicity towards *H. incongruens* than for the test plants. These outcomes are in agreement with the results of other research in which higher toxicity of heavy metal-polluted soils was identified towards invertebrates than for plants (Szara et al. 2020; Wieczorek and Baran 2021). The higher sensitivity of *H. incongruens* results from the fact that this organism is in direct contact with the medium (i.e., soil) and that the main exposure route is the oral route (De Cooman et al. 2015; Heise et al. 2020). Besides, the lower sensitivity of test plants (Phytotoxkit test) may be related to the lower sensitivity of plants towards heavy metals than in the case of invertebrates (Ostracodtoxkit) (Wieczorek and Baran 2021). It may result from one of the most important protective mechanisms of plants which is related to efficient metal detoxication in the root cells and thus limited translocation of metals through the xylem to the aboveground parts (Wierzbicka et al. 2007). The low toxicity of investigated soil samples towards living organisms may result from two main reasons. First, it is possible that the application of soil amendments resulted in the immobilization of toxic elements in the soil. Secondly, an increase in the uptake of toxic elements was noted in the soil mixed with highly contaminated post-industrial soil (object 4).

## Conclusions

1. The field experiment was performed on the Municipal Waste Rendering Plant soil, characterized by high salinity, with the content of heavy metals below the permissible limits established for post-industrial soils.
2. The highest plant yield was obtained for the soil mixed with 400 Mg‧ha^−1^ of SK-8 (object 3).
3. The highest concentration of Zn, Cr, Cu, and Mn was observed for giant miscanthus cultivated on the Municipal Waste Rendering Plant soil (object 1); meanwhile, the primary source of Al, Fe, Pb, Co, and Ni was SK-8. In the case of cadmium accumulation in the plant biomass, the highest content was measured for plants cultivated on soil mixed with highly contaminated post-industrial soil (object 4).
4. The highest uptake of Zn, Cr, and Cu by plants was observed for object 1. Incorporation of SK-8 into the soil (objects 2 and 3) led to an increase in the uptake of Al, Fe, Co, Pb, Mn, Ni, and Cd in comparison to object 1. Object 4 favorably affects the uptake of Al, Fe, Co, Pb, Mn, Ni, and Cd by the plant in comparison to object 1.
5. With the dose of SK-8 equal to 400 Mg‧ha^−1^, the amount of elements introduced to the soil environment was the highest. Furthermore, in this object, the largest balance difference was noticed.
6. The phytorecovery of the elements did not exceed 1% of the amount occurring in the soil. This fact resulted from a short cultivation period and large doses of SK-8 or highly contaminated post-industrial soil. The greatest phytorecovery was obtained for Cu (0.755%), next in order Zn, Ni, Mn, Cr, Co, Fe, Cd, Pb, and the lowest amount of Al (0.019%).
7. After the first year of vegetation, giant miscanthus showed good ability to accumulate contaminants, which indicates that this species can be successfully used for the biological treatment of post-industrial soils, especially in landfills and settling tanks with different levels (i.e., wide range) of pollution.

## Data Availability

All data generated during the current study are included in this published article.
