# Correction to: Remediation of soils on municipal rendering plant territories using *Miscanthus* × *giganteus*

**DOI:** 10.1007/s11356-025-36532-y

**Published:** 2025-05-16

**Authors:** Anna Grzegórska, Natalia Czaplicka, Jacek Antonkiewicz, Piotr Rybarczyk, Agnieszka Baran, Krzysztof Dobrzyński, Dawid Zabrocki, Andrzej Rogala

**Affiliations:** 1 Department of Process Engineering and Chemical Technology, Faculty of Chemistry, Gdansk University of Technology, Narutowicza 11/12, 80-233 Gdansk, Poland; 2 Department of Agricultural and Environmental Chemistry, Faculty of Agriculture and Economics, University of Agriculture in Krakow, Av. Mickiewicza 21, 31-120 Krakow, Poland; 3 Rendering Plant in Gdańsk, Zakład Utylizacyjny Sp. z o.o. w, Jabłoniowa 55, 80-180 Gdansk, Poland; 4 Research and Development Dawid Zabrocki, Jęczniki Wielkie 36 A, 77-300 Czluchow, Poland

**Correction to: Environmental Science and Pollution Research (2023) 30:22305–22318**

10.1007/s11356-022-23724-z

**Acknowledgement** This manuscript was prepared within the scope of the Baltic Phytoremediation (BAPR) project, co-financed by the Interreg South Baltic Program 2014–2020 under project number STHB.02.02.00-SE-0155/18, and published as part of an international project co-financed by the program of the Minister of Science and Higher Education entitled “PMW” in 2021-2022-Baltic Phytoremediation (BAPR) under contract no. 5247/SBP 2014-2020/2021/2.

